# Interaction of *legionella pneumophila *and *helicobacter pylori *with bacterial species isolated from drinking water biofilms

**DOI:** 10.1186/1471-2180-11-57

**Published:** 2011-03-18

**Authors:** Maria S Gião, Nuno F Azevedo, Sandra A Wilks, Maria J Vieira, Charles W Keevil

**Affiliations:** 1 School of Biological Sciences, Life Sciences Building, Highfield Campus, University of Southampton, Southampton SO17 1BJ, UK; 2 Institute for Biotechnology and Bioengineering, Centre of Biological Engineering, Universidade do Minho, Campus de Gualtar 4710-057, Braga, Portugal; 3 LEPAE, Department of Chemical Engineering, Faculty of Engineering, University of Porto, Porto, Portugal

## Abstract

**Background:**

It is well established that *Legionella pneumophila *is a waterborne pathogen; by contrast, the mode of *Helicobacter pylori *transmission remains unknown but water seems to play an important role. This work aims to study the influence of five microorganisms isolated from drinking water biofilms on the survival and integration of both of these pathogens into biofilms.

**Results:**

Firstly, both pathogens were studied for auto- and co-aggregation with the species isolated from drinking water; subsequently the formation of mono and dual-species biofilms by *L. pneumophila *or *H. pylori *with the same microorganisms was investigated. Neither auto- nor co-aggregation was observed between the microorganisms tested. For biofilm studies, sessile cells were quantified in terms of total cells by SYTO 9 staining, viable *L. pneumophila *or *H. pylori *cells were quantified using 16 S rRNA-specific peptide nucleic acid (PNA) probes and cultivable cells by standard culture techniques. *Acidovorax *sp. and *Sphingomonas *sp. appeared to have an antagonistic effect on *L. pneumophila *cultivability but not on the viability (as assessed by rRNA content using the PNA probe), possibly leading to the formation of viable but noncultivable (VBNC) cells, whereas *Mycobacterium chelonae *increased the cultivability of this pathogen. The results obtained for *H. pylori *showed that *M*. *chelonae *and *Sphingomonas *sp. help this pathogen to maintain cultivability for at least 24 hours.

**Conclusions:**

It appears that *M. chelonae *may have an important role in the survival of both pathogens in drinking water. This work also suggests that the presence of some microorganisms can decrease the cultivability of *L. pneumophila *but not the viability which indicates that the presence of autochthonous microorganisms can lead to misleading results when the safety of water is assessed by cultivable methods alone.

## Background

In natural environments, bacteria can adhere to surfaces forming a complex structure called a biofilm. When embedded in biofilms, microorganisms can be protected from several adverse factors such as temperature, low nutrients and the presence of biocides [1-6]. Therefore, understanding the ecology of microorganisms in this structure is fundamental in order to obtain a comprehensive knowledge of real systems. In nature, biofilms typically consist of many species of microorganisms that can interact with each other either positively (for instance, the synthesis of a metabolite by one species which can be used in the metabolism of another) or negatively (such as nutrient competition) [7-9]. One type of biofilm that has been widely studied is that formed in drinking water distribution systems (DWDS) because of its role in the persistence of pathogens in drinking water and the consequent potential for impact on public health [10-12].

*Legionella pneumophila *is a waterborne pathogen that can cause Legionnaires' disease or Pontiac fever [13,14]. This pathogen is found naturally in fresh water reservoirs and can contaminate drinking water when disinfection is inefficient, being transmitted to man when contaminated aerosols are inhaled [12,15-17]. The mode of transmission of *Helicobacter pylori *remains controversial but drinking water as a route of transmission has recently gained recognition [18]. Although no cultivable *H. pylori *have ever been recovered from drinking water systems, molecular techniques such as PCR [19-22] and peptide nucleic acid (PNA) probes used to target 16 S rRNA in fluorescence *in situ *hybridization (FISH) assays [23,24], have demonstrated the presence of this pathogen in DWDS. This identification, in addition to epidemiological studies, point to different prevalence of *H. pylori *in the microbial population which is associated with the type of source water. This strongly supports water as a route of transmission [18,25-27].

Previous studies have demonstrated that both pathogens can be incorporated into heterotrophic drinking water biofilms and persist for at least 32 days [28,29]. In the case of *H. pylori*, although no cultivable cells were ever recovered, the presence of a high intracellular rRNA content indicates that cells might be in a viable but non cultivable (VBNC) state [28,30]. It is possible that the loss of *H. pylori *cultivability when associated with heterotrophic biofilms had been due to a negative effect caused by the presence of other microorganisms [31]. Nevertheless, it is also possible that there were other microorganisms present in the biofilm that could have a beneficial effect on *L. pneumophila *or *H. pylori*, as shown by other studies where these pathogens were co-cultured with other microorganisms in liquid media [32,33]. However, for multi-species biofilms it is technically very challenging to determine which sessile microorganisms could have a positive or negative effect on these pathogens, particularly regarding the intimate associations that occur within biofilms. A particular type of interaction that can facilitate the formation of biofilm is the aggregation of cells, which can occur between cells of the same species (auto-aggregation) or between different species (co-aggregation), and has been well described for isolates of dental plaque species in complex media and aquatic species in potable water [34-36].

The aim of this work was to study the influence of different autochthonous microorganisms isolated from drinking water biofilms on the incorporation and survival of *L. pneumophila *and *H. pylori *in biofilms. For that, the first part of the work tested all the species used for auto and co-aggregation. Subsequently, dual-species biofilms of *L*. *pneumophila *and *H. pylori *were formed with the different drinking water bacteria and the results compared with mono-species biofilms formed by *L. pneumophila *and *H*. *pylori*.

## Results

### Auto and co-aggregation of *L. pneumophila *and other drinking water bacteria

Initially, the selected biofilm strains were tested for auto- and co-aggregation in test tubes as described by Rickard et al. [35], either alone or with *L. pneumophila*. No co-aggregation was observed for the strains studied, either alone or in pairs with *L*. *pneumophila *(results not shown).

### *L. pneumophila *in biofilms

For the experiments on biofilm formation on uPVC coupons, an inoculum of *L*. *pneumophila *was prepared containing approximately 3.7 × 10^7 ^of total cells ml^-1 ^(quantified using SYTO 9 staining). In comparison to total cells, 49% were cultivable on BCYE agar and 50% were detected by PNA-FISH. The inocula of the strains isolated from drinking water biofilms had on average 75% of cultivable cells compared to SYTO 9 stained cells, except in the case of *Mycobacterium chelonae *where the percentage was considerably lower (2.5%).

**Figure 1:**
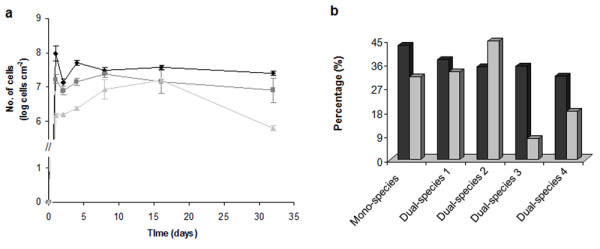
**Numbers of *L. pneumophila* cells in mono and dual-species biofilms**. Variation of the number of cells of *L. pneumophila *in mono-species biofilm quantified by the three different methods: curves represent the variation of total cell number (black diamond), *L. pneumophila *hybridized with the PNA PLPEN620 probe (dark grey square) and cultivable *L. pneumophila *(light grey triangle); bars represent standard deviation (n = 3) (a). *L. pneumophila *PNA-positive numbers/total cells numbers ratio (dark grey bars) and cultivable *L. pneumophila *numbers/*L. pneumophila *PNA-positive numbers ratio (light grey bars) for the mono-species biofilm and dual-species biofilms of *L. pneumophila *and *V. paradoxus *(Dual-species 1), *L. pneumophila *and *M*. *chelonae *(Dual-species 2), *L. pneumophila *and *Acidovorax *sp. (Dual-species 3) and *L. pneumophila *and *Sphingomonas *sp. (Dual-species 4); the ratio values were calculated using the average of the values obtained for the six time point samples (b).

Figure 1a shows the variation with time of total cells, PNA-cells and cultivable *L*. *pneumophila *present in a mono-species biofilm. The attachment of *L. pneumophila *cells to the surface occurred in the first 24 hours of the experiment. Moreover, the numbers of total cells (stained by SYTO 9) and PNA stained cells did not change significantly between days 1 and 32 (P > 0.05). In contrast, the number of cultivable cells greatly increased in the first two weeks and then decreased significantly in the last weeks of the experiments (P < 0.05).

**Figure 2:**
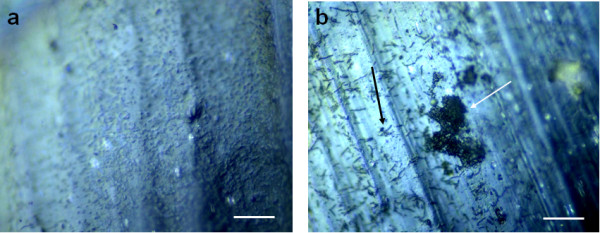
**uPVC coupon covered with a mono and dual-species *L. pneumophila* biofilm.** Microphotograph of an uPVC coupon visualized under EDIC microscopy covered with a 32 days-old biofilm formed by *L. pneumophila *(a) and *L. pneumophila *and *Sphingomonas *sp. (b). The black arrow indicates individual cells attached to the uPVC surface and white arrow indicates a microcolony. Bars represent 20 μm.

For the experiments of *L. pneumophila *in dual-species it was observed that the numbers of *L. pneumophila *PNA-positive cells and cultivable *L. pneumophila *did not change significantly with time after the first day (P > 0.05). Table 1 presents the data obtained for the quantification of sessile cells, giving the average values of the samples analyzed at all time points, for mono and dual-species biofilms. The data for the numbers of total cells, total PNA-positive *L. pneumophila *and cultivable *L. pneumophila *in mono and in dual species biofilms were similar (P > 0.95), except for the numbers of cultivable *L*. *pneumophila *when associated with *Acidovorax *sp. which were significantly lower (P < 0.05). Figure 1b shows the percentage of PNA-positive *L. pneumophila *in relation to SYTO 9 stained total cells; this was similar for both mono and dual-species biofilms (P = 1.000). This indicates that *L. pneumophila *adhere well to uPVC surfaces, either alone or in the presence of *Variovorax paradoxus*, *M. chelonae*, *Acidovorax *sp. And *Sphingomonas *sp., although the morphology of the biofilm appeared to be different for the mono or dual-species (Figure 2a and 2b, respectively). The relationship between cultivable and *L. pneumophila *PNA-positive cells was higher (although not statistically significant, P > 0.95) for cells recovered from the *L. pneumophila *- *M. chelonae *biofilm while the numbers of cultivable *L. pneumophila *decreased five-fold when this pathogen was associated with *Acidovorax *sp. and almost four-fold when associated with *Sphingomonas *sp.

**Table 1:** Average of the total number of cells, *L. pneumophila *PNA-positive, cultivable *L. pneumophila *and cultivable non-*legionellae *cell numbers in mono and dual-species biofilms obtained for all the time points sampled.

Strain in biofilm	Total cells × 10^-7 ^(cells cm^-2^)	PNA cells × 10^-7 ^(cells cm^-2^)	Cultivable *L. pneumophila *× 10^-6 ^(CFU cm^-2^)	Cultivable non - *legionellae *× 10^-6 ^(CFU cm^-2^)
*L. pneumophila*	4.42	1.48	5.25	n.a.

*L. pneumophila *and *V. paradoxus*	3.51	1.11	4.11	4.49
*M. chelonae*	4.87	1.05	4.65	0.19
*Acidovorax sp*.	4.12	1.59	1.05	6.55
*Sphingomonas sp*.	3.80	0.83	1.45	1.06

n.a. - not applicable.

### Auto and co-aggregation of *H. pylori *and other drinking water bacteria

The same experiments were repeated using *H. pylori *instead of *L. pneumophila*. For the auto- and co-aggregation of *H. pylori *with drinking water isolates, the same strains were used as selected for the *L. pneumophila *experiments and an additional strain was also included: *Brevundimonas *sp., a bacterium isolated on CBA medium from drinking water biofilms. The results obtained in the test tube assay system showed neither auto nor co-aggregation of *H. pylori *with any of the species investigated.

### *H. pylori *in biofilms

**Figure 3:**
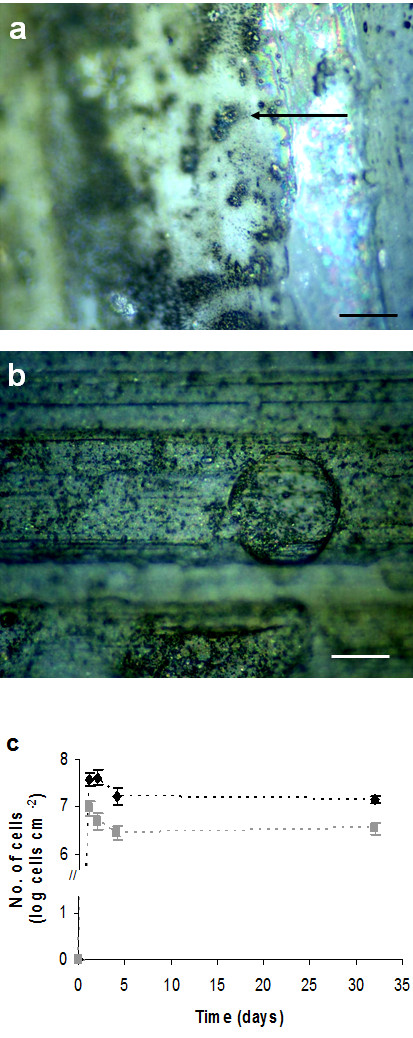
**uPVC coupon covered with *H. pylori* biofilm and variation of *H. pylori *numbers in the mono-species biofilm**. Microphotograph of an uPVC coupon visualized under EDIC microscopy covered with a mono-species *H. pylori *biofilm after 1 day (a) and 32 days (b) of incubation. Black arrow indicates the presence of a microcolony. Bars represent 20 μm. (c) Variation with time in the total cell number (black diamond) and *H. pylori *PNA-cells (grey square) present in the biofilm. Bars represent standard deviation (n = 3).

The biofilm experiments used the same strains indicated in the previous paragraph. It was observed that for the *H. pylori *inoculum, only 5% of the total cells were cultivable, a value similar to that obtained by Azevedo et al. [37], while 29% were detected by PNA-FISH. Figure 3a and 3b show that *H. pylori *is able to form biofilms, despite the poor cultivability of the cells on agar media. However, while the morphology of *H*. *pylori *cells from the inoculum was predominantly spiral, after forming biofilms the cells were mainly coccoid shaped.

Figure 3c shows that when in pure culture *H. pylori *adhered to the surface to form the biofilm in the first day followed by a statistically significant decrease (P < 0.05) in total cells during day 1 and 4. The same trend was observed for cells quantified using the PNA probe. No cultivable *H. pylori *were recovered on CBA medium. When the biofilm was formed in the presence of *Brevundimonas *sp. the variation with time of total cells and PNA numbers were not statistically significant (P > 0.05). Comparing the numbers obtained for pure *H. pylori *biofilms and biofilms grown in the presence of *Brevundimonas *sp. there was no significant difference between the numbers of *H. pylori *detected using the PNA probe (results not shown) nor in the percentage between PNA and total cells numbers (P > 0.05).

In terms of cultivable cells it was observed that no cultivable *H. pylori *were ever recovered from any of the mono or dual-species biofilms at any time point, with the exception of cells recovered from 1 day-old biofilms grown in the presence of *M*. *chelonae *or *Sphingomonas *sp. (6.67 × 10^1 ^and 1.83 × 10^2 ^CFU cm^-2^, respectively).

## Discussion

### Auto and co-aggregation of *L. pneumophila *and *H. pylori *with drinking water bacteria

In a previous study several bacterial strains were isolated from heterotrophic biofilms formed on uPVC coupons in a two-stage chemostat system [28]. For the present work, the selection of the bacteria used was based on the prevalence of these isolated strains in biofilms, i.e., the strains that were always present in biofilm samples when detected by culture were used rather than those only found intermittently.

In the aggregation studies it was observed that there was no auto-aggregation of any of the bacteria tested in this study, as demonstrated previously for *Brevundimonas vesicularis*, *Acidovorax delafieldii *and *V. paradoxus *[34,38]. No co-aggregation of *L. pneumophila *or *H. pylori *was observed with any of the bacteria isolated from drinking water biofilms, demonstrating that while all of the bacteria used in this study have the ability to form biofilms they are attaching to the uPVC surfaces without aggregating in the planktonic phase with the other microorganisms [36].

### *L. pneumophila *in biofilms

The *L. pneumophila *cells from the inocula prepared for the biofilm experiments were quantified for total, PNA-positive and cultivable cells. Results showed that cultivable and PNA numbers were similar but were only 50% of the numbers obtained by SYTO 9 staining. It is still controversial whether PNA probes detect dead cells or if they just produce a detectable signal with viable cells. PNA probes have been used to detect pathogens in mixed biofilms but it has not been well established if this technique can also detect non-viable cells [23,29,39]. However the similarity in the cultivable and PNA-positive numbers, and the difference between PNA-labelled and total cells (stained by SYTO 9), strongly indicates that the PNA probe fails to detect dead cells. PNA probes bind specifically to rRNA molecules emitting a signal that can be visualized under microscopy. The intensity of that signal is related to the rRNA content, i.e., the higher the rRNA content the brighter the signal is [40]. A very low content of rRNA would result in insufficient brightness and cells would not be visualized. After cellular death the content of rRNA decreases significantly and therefore some authors have suggested that the emission of a bright signal is a good indication of cell viability [39,41,42]. The results obtained in the present study (Figure 1a) support that there is a correlation between the number of viable cells and the number of cells that bind to the PNA probe but further studies should be performed to correlate the PNA-positive cells and their metabolic state.

As it has been demonstrated before by other authors [43,44], the attachment of *L*. *pneumophila *cells to the uPVC surface occurred on the first day of biofilm formation and the numbers of total and PNA stained cells, from mono-species biofilms, did not change significantly (P > 0.05). Nevertheless, the numbers of cultivable cells increased in the first two weeks and decreased during the rest of the experiment. It has been demonstrated that *L. pneumophila *can survive in tap water for long periods without losing cultivability [45,46], but is not able to replicate in axenic cultures in tap water or in low nutrient media, except when associated with biofilms or parasitizing amoebal species [29,47,48]. After two weeks the cultivability decreased but was not completely lost for the 32 days of the experiment which indicates that biofilms are a protective niche for *L. pneumophila*, even in axenic culture. Conversely, PNA-positive numbers with a high fluorescence intensity remained constant and, for the same reason explained before, this suggests that cells are still viable. Moreover, the fact that total *L*. *pneumophila *and *L. pneumophila *PNA-positive cells remained constant with time indicates that there is no damage to DNA and rRNA, respectively. Conversely, the variation of PNA-positive numbers in dual-species biofilms was used as an indicator of the variation of viable *L. pneumophila *cells inside of those biofilms.

The results of dual-species biofilms showed that when biofilms were formed in the presence of *M. chelonae *the percentage of cultivable *L. pneumophila *in relation to *L*. *pneumophila *PNA-positive cells was slightly superior compared to mono-species biofilms or dual-species biofilms with the other strains isolated from drinking water. Although the difference is not statistically significant this result indicates that this strain has a small positive effect on *L. pneumophila *cultivability. In contrast, the numbers of cultivable *L. pneumophila *decreased when this pathogen was associated with *Acidovorax *sp. indicating that this species has a negative impact on *L. pneumophila *cultivability. It was also observed that the numbers of cultivable *L. pneumophila *when co-cultivated with *Sphingomonas *sp. decreased and, although the statistical analysis showed that the difference is not significant, the fact that the cultivability was almost four-fold lower appears to reveal an antagonistic effect. Conversely, it appears that both strains affect negatively sessile *L. pneumophila *cultivability, either by competition for nutrients or production of a metabolite toxic to *L. pneumophila*. The fact that these two species were isolated on R2A reveals that they have low nutritional requirements to grow and might even be able to grow in water, contrary to *L. pneumophila *which is not able to grow in absence of, for example, L-cysteine and high iron concentrations [49,50]. This is corroborated by the values shown in Table 1, where cultivable *Acidovorax *sp. and *Sphingomonas *sp. numbers are 6.55 × 10^6 ^and 1.06 × 10^6 ^CFU cm^-2 ^suggesting that these two microorganisms could be metabolically active in the biofilm despite the poor nutrient concentration of the medium (filtered tap water). Another possible explanation for the lower numbers of cultivable *L. pneumophila *when biofilms were formed in co-culture with *Sphingomonas *sp., can be related to the structure of the biofilm. Figure 2 shows a 32 days-old biofilm formed by *L. pneumophila *and *L*. *pneumophila *associated with *Sphingomonas *sp. The biofilm formed in the presence of *Sphingomonas *sp. had a different morphology, and although the thickness of the biofilm has not been measured, the presence of microcolonies suggests the presence of thicker structures where anaerobic zones might occur. Wadowsky et al. [33] have demonstrated that in anaerobic conditions *L. pneumophila *loses cultivability and if biofilms formed by *L. pneumophila *and *Sphingomonas *sp. have indeed anaerobic zones, then it is possible that *L. pneumophila *located in those places has become uncultivable. It would therefore be interesting to undertake further research to measure the thickness of different parts of the biofilm and the respective concentration of oxygen and relate those results to the cultivability of cells from those regions. However, the fact that the numbers quantified by the use of a PNA probe remained constant, might indicate that these cells may still be viable and can probably recover cultivability in favorable conditions.

This work clearly demonstrates that *L. pneumophila *can be negatively or positively influenced by other microorganisms present in drinking water. It is important to note that this study was carried out under particular conditions and it will be important to perform more experiments in the future, in particular to study the effect of other drinking water bacteria, the formation of biofilms under dynamic conditions and the incorporation of a disinfectant, such as chlorine.

It is known that other bacteria can influence the growth of *L. pneumophila *either in nutrient-poor environments, such as drinking water, or in rich artificial media. Toze et al. [51] have demonstrated that some bacteria commonly present in heterotrophic biofilms, such as *Pseudomonas *sp. and *Aeromonas *sp., can inhibit the growth of *L*. *pneumophila *while Wadowsky and Yee [49] demonstrated that *Flavobacterium breve *can support the satellite growth of this pathogen on BCYE agar without L-cysteine. A curious result was obtained by Temmerman et al. [52] who demonstrated that dead cells can also support the growth of this pathogen. Although the mechanisms responsible for the influence of different microorganisms on *L. pneumophila *survival are unknown there is one aspect of *L. pneumophila *microbial ecology that has been already well-established: *L. pneumophila *is not able to grow in drinking water unless associated with biofilms or amoebal species [46,47,53]. Hence, the knowledge of how microorganisms affect *L. pneumophila *cultivability is a key factor for the effective control of this pathogen in drinking water and associated biofilms, and requires further investigation.

### *H. pylori *in biofilms

In this study the cells recovered from mono-species *H. pylori *biofilms were always uncultivable, for all the time points, which is in contrast to the Azevedo et al. [54] study, where it was demonstrated that after 24 hours sessile *H. pylori *cells were still cultivable. This might be due to the differences in the method of cell removal from the coupons, the quality of water or the type of uPVC substratum. When the biofilm was formed in the presence of *Brevundimonas *sp. no cultivable *H. pylori *cells were ever recovered either. However, for this case, care should be taken in the interpretation of the results. In fact, *Brevundimonas *was able to grow on CBA medium in a faster and more abundant way then *H. pylori*. As such, it is impossible to determine whether *H. pylori *is indeed uncultivable in the presence of this microorganism, or whether it could not be detected because it was overgrown by *Brevundimonas*. We have attempted to solve this issue by using CBA medium supplemented with antibiotics but, as shown by other authors [28], available selective medium for *H. pylori *allows the growth of other species, including *Brevundimonas *sp. The fact that there were no differences in the results for the PNA-positive cell numbers obtained for *H. pylori *in mono-species biofilms and in dual-species biofilms with *Brevundimonas *sp. suggests that this bacterium has little or no effect on the inclusion of *H. pylori *in biofilms.

Cultivable *H. pylori *was never recovered from dual-species biofilms at any time point, independently of the second species used, except when *H. pylori *formed dual-species biofilms in the presence of *M. chelonae *and *Sphingomonas *sp. For these two microorganisms, it was observed that *H. pylori *was able to retain cultivability for a period of between 24 and 48 hours. This suggests that both microorganisms might have a positive effect on the inclusion and survival of this pathogen in drinking water biofilms. The ability of *H. pylori *to adapt to different physico-chemical parameters has been studied by several authors [30,55-58], however no studies about the influence of other microorganisms on the survival of this pathogen have been found in the literature except the coculture of *H. pylori *with the protozoan, *Acanthamoeba castellanii *[59]. The interaction of microorganisms in biofilms has been widely studied and in this particular case could be the key for the survival of this microorganism in drinking water systems, even if in a VBNC state. More investigations should therefore be performed concerned with the influence of drinking water microorganisms on *H. pylori *metabolism and survival.

## Conclusions

This work clearly demonstrates that, even in pure culture, both pathogens can adhere to surfaces and form biofilm. *L. pneumophila *can remain cultivable for at least 32 days although less cultivable when associated with *Acidovorax *sp. and *Sphingomonas *sp. The experiments with *H. pylori *demonstrated that this pathogen loses cultivability in less than 24 hours when in mono-species or in dual-species biofilms with *V. paradoxus*, *Acidovorax *sp. and *Brevundimonas *sp., while retaining cultivability for at least 24 hours when biofilms are grown in the presence of *M. chelonae *and *Sphingomonas *sp. Consequently, *M. chelonae *seems to have a positive effect on the cultivability of both pathogens and being a pathogen commonly found in drinking water systems [60,61], can play an important role in the control of these two pathogens. Control of this mycobacterial opportunistic pathogen and other biofilm species that can have a synergetic effect on *L. pneumophila *and *H. pylori *might provide an important contribution towards the supply of safe drinking water as both *L. pneumophila *and *H*. *pylori *have been found to be chlorine resistant [62,63].

## Methods

### Culture maintenance

In this work, *L. pneumophila *NCTC 12821 and *H. pylori *NCTC 11637 strains were used. Strains of *V. paradoxus*, *M. chelonae*, *Acidovorax *sp., *Sphingomonas *sp. and *Brevundimonas *sp. were isolated from drinking water biofilms [28,29]. All strains were maintained in vials frozen at -80°C and recovered by standard plating procedures onto the appropriate media and subcultured once prior to biofilm formation experiments. *L*. *pneumophila *NCTC 12821, *V. paradoxus *and *M. chelonae *were grown on Buffered Charcoal Yeast Extract (BCYE) agar (Oxoid, UK) for 24 hours at 30°C. *Acidovorax *sp. and *Sphingomonas *sp. were grown on R2A (Oxoid, UK) for 48 hours at 22°C. *H. pylori *NCTC 11637 and *Brevundimonas *sp. were grown on Columbia Agar (Oxoid, UK) supplemented with 5% (v/v) defibrinated horse blood (CBA) (Oxoid, UK) and incubated for 48 hours at 37°C in a microaerophilic atmosphere of 10% CO_2_, 7% H_2 _and 3% O_2 _(the remainder being N_2_).

### Auto- and co-aggregation in test tubes

Prior to the start of the experiments tap water from Southampton, UK, was collected in a transparent flask and left, loosely closed, overnight for chlorine evaporation. Then the water was sterilized by filtration through a 0.2 μm pore size Nylon filter (Pall Gelman, UK). All bacterial species were suspended in this dechlorinated and filtered tap water, with the following characteristics, provided by the water company (Southern Water, UK): pH 7.3; turbidity 0.10 FTU; conductivity 504 μS cm^-1^; total organic carbon 0.649 mg l^-1^; total iron 16 μg Fe l^-1^; free chlorine 0.21 mg Cl_2 _l^-1^; total chlorine 0.26 mg Cl_2 _l^-1^. The inocula had a final concentration of approximately 2 × 10^8 ^cells ml^-1^. For autoaggregation, 3 ml of each suspension was transferred into a sterile test tube, whereas for co-aggregation experiments 1.5 ml of either *L. pneumophila *or *H. pylori *suspension were mixed with 1.5 ml of each one of the species isolated from drinking water biofilms. At times 0, 1, 2, 4, 6, 8, 24 and 48 hours, tubes were vortexed for 10 seconds and observed for co-aggregation according to the scale described by Rickard et al. [35]. All experiments were performed in duplicate.

### Coupon preparation

Unplasticized polyvinylchloride (uPVC) coupons of 1 cm^2 ^were used as a substratum for biofilm growth as it is a commonly used material in drinking water pipelines. To remove grease and wax from the coupons, prior to biofilm growth, they were immersed in water and detergent for 5 min, washed with a bottle brusher, rinsed twice in distilled water and air-dried. Subsequently, they were washed in 70% (v/v) ethanol to remove any organic compounds and autoclaved at 1 atm and 121°C [64].

### Biofilm formation

To form the mono-species biofilms of *L. pneumophila *NCTC 12821 and *H. pylori *NCTC 11637 the inocula were prepared by suspending the cells in 50 ml of dechlorinated and filtered tap water to give a final concentration of approximately 10^7 ^cells ml^-1^. The mono-species biofilms were used as a control. The dual-species biofilm inocula were prepared by mixing *L. pneumophila *or *H. pylori *with *V. paradoxus*, *M*. *chelonae*, *Acidovorax *sp. or *Sphingomonas *sp. in 50 ml of filter-sterilized tap water to a final concentration of 10^7 ^cells ml^-1 ^of each microorganism. For the experiments with *H*. *pylori *an inoculum was also prepared with this pathogen and *Brevundimonas *sp. All suspensions were homogenized by vortexing and 4 ml of each inoculum were transferred to 6-well microtiter plates containing one uPVC coupon in each well. Plates were incubated in the dark at 22°C and two coupons of each biofilm type were removed after 1, 2, 4, 8, 16 and 32 days, and gently rinsed to remove loosely attached cells on the surface of the biofilm. One coupon was used for direct observation under a Nikon Eclipse E800 episcopic differential interference contrast/epifluorescence (EDIC/EF) microscope (Best Scientific, UK) [65] using the EDIC channel to directly visualise biofilm. The other coupon was scraped to quantify sessile cells.

### Quantification of sessile cells

At each time point coupons were removed from the wells and rinsed three times in filtered tap water to remove planktonic cells from the biofilm and coupons surfaces. The coupons were then transferred to a 15 ml centrifuge tube (Greiner Bio-one, UK) containing 2 ml of filter-sterilized tap water and autoclaved glass beads of 2 mm diameter (Merck, UK). To remove the biofilm from the coupon surfaces the tubes were then vortexed for 1 min. The vortexing step also promoted the homogenization of the suspensions prior to the quantification of total cells, PNA-positive cells and cultivable cells, as described below. Preliminary experiments showed that vortexing with glass beads removed the biofilm formed under these conditions, although it was still possible to observe a few dispersed cells on the uPVC surface.

Total cells were quantified using the SYTO 9 staining method (Molecular Probes, Invitrogen, UK). In summary, 1 ml of an appropriate dilution was mixed with 0.5 μl of SYTO 9, incubated in the dark for 15 minutes, filtered through a 0.2 μm pore size polycarbonate black Nucleopore^® ^membrane (Whatman, UK) and allowed to air-dry. Then a drop of non-fluorescent immersion oil (Fluka, UK) and a coverslip were added before observation under the Nikon Eclipse E800 EDIC/EF microscope (Best Scientific, UK) [65]. As the cells were homogenously distributed, 10 fields of view on each membrane were chosen at random and the number of cells counted (×100 objective lens).

*L. pneumophila *was quantified using the specific PNA probe PLPNE620 (5'-CTG ACC GTC CCA GGT-3') and *H. pylori *by the use of a PNA probe with the following sequence 5'- GAGACTAAGCCCTCC -3'(Eurogentec, Belgium). PNA-FISH was carried out by filtering 1 ml of an appropriate dilution through a 0.2 μm Anodisc membrane (Whatman, UK). This was left to air dry. For the quantification of *L*. *pneumophila *the membrane was covered with 90% (v/v) ethanol to fix the cells and again air dried. The hybridization, washing and microscopy observation method was performed as described by Wilks and Keevil [42]. For *H. pylori *quantification the membrane was covered with 4% (w/v) paraformaldehyde followed by 50% (v/v) ethanol for 10 minutes each to fix the cells and air dried. The hybridization, washing and microscopy observation method was performed as described by Guimarães et al. [66].

Cultivable numbers of all bacteria were determined by plating 40 μl of an appropriate dilution on the respective agar medium, as described above in the section "Culture maintenance". BCYE plates were incubated aerobically for two days at 30°C, R2A for seven days at 22°C and CBA plates were incubated for seven days at 37°C in a microaerophilic atmosphere. It is recommended that the incubation of BCYE to quantify *L. pneumophila *from environmental samples goes for up to ten days. However it was observed that for these samples if the BCYE plates were incubated for more than two days the colonies would overgrow in diameter and it would be impossible to distinguish individual colonies. Therefore two days was chosen as the incubation time.

### Statistical analysis

The homogeneity of variances of total number, PNA and cultivable cells and the relation between *L. pneumophila *of cells and total cells was checked by the Levene test for equality of variances using a statistical package (SPSS Inc., Chicago IL, USA). Results were subsequently compared by a one-way ANOVA followed by a Bonferroni *post hoc *test. Differences were considered relevant if P < 0.05.

## Authors' contributions

MSG participated in the experimental design, carried out all experimental work and drafted the manuscript. NFA, SAW, MJV and CWK participated in the design of the study and helped to draft the manuscript. All authors have read and approved the final manuscript.

## Aknowledgements

This work was supported by the Portuguese Institute Fundação para a Ciência e Tecnologia (PhD grant SFRH/BD/17088/2004 and post-doc grant SFRH/BPD/20484/2004).
